# Synthesis of Amphiphilic Polyether-Modified Silicone Oil Polymers and the Application of Their Micelles in Enhancing the Overall Waterproofing and Corrosion Resistance of Cement-Based Concrete Materials

**DOI:** 10.3390/polym18101153

**Published:** 2026-05-08

**Authors:** Yujie Luo, Fen Zhou, Shuangping Ma, Depeng Gong, Zhanbo Wang, Xi Li, Chaocan Zhang

**Affiliations:** 1 School of Materials Science and Engineering, Wuhan University of Technology, Wuhan 430070, China; lyj04150926@163.com (Y.L.); 245184@whut.edu.cn (F.Z.); 13807123638@139.com (S.M.); gdp@whut.edu.cn (D.G.); wzb159200@163.com (Z.W.); 2 School of Chemistry, Chemical Engineering and Life Science, Wuhan University of Technology, Wuhan 430070, China; chemlixi@whut.edu.cn

**Keywords:** amphiphilic polyether-modified silicone oil micelles, hydrophobic admixture, cement-based materials, waterproof performance, corrosion resistance

## Abstract

In this study, a series of amphiphilic polyether-modified silicone oil (PMSO) polymers with hydrophilic–lipophilic balance (HLB) values ranging from 3.5 to 5 were synthesized via hydrosilylation. These polymers are self-emulsifying and can form stable micelles in water without the need for external emulsifiers, with micelle sizes ranging from 79.9 to 161.4 nm. For the first time, such amphiphilic micelles were employed as an internally incorporated hydrophobic admixture to investigate the waterproofing and corrosion resistance of cement-based materials. The results showed that PMSO micelles with an HLB value of 4 significantly reduced the water absorption of cement mortar and improved mechanical properties by enhancing the compactness and crystallinity of the cement matrix. At a dosage of 0.5 wt% PMSO in mortar, the water absorption at 48 h was reduced by 50.27% compared with the control group, and the inner and outer contact angles of the mortar specimens reached 105° and 126°, respectively. The chloride diffusion coefficient of concrete decreased by 70.8% relative to the control group. At an appropriate dosage (0.1 wt%), the flexural strength of the mortar at 28 days increased by 12.50%, and the compressive strength increased by 14.19% compared with the control group. Low-field nuclear magnetic resonance (NMR) was used to determine the changes in the pore structure of mortar specimens before and after the addition of PMSO micelles. The experimental results showed that the addition of PMSO micelles reduced the number of harmful large pores, resulting in a denser microstructure. Finally, the corrosion resistance of concrete was evaluated via electrochemical accelerated-corrosion aging tests. The cracking time of concrete containing PMSO micelles was extended from 144 h (control group) to 240 h, demonstrating improved corrosion resistance.

## 1. Introduction

Due to its excellent performance and low cost, cement concrete has become the most widely used civil engineering material in the world and serves as the material foundation for human development [1]. However, because cement-based materials have a porous structure and are hydrophilic, harmful substances such as chloride ions, sulfate ions, and hydrogen ions can easily penetrate the concrete through water, leading to the degradation of concrete structures and the corrosion of reinforcing steel [2,3]. At the same time, CO_2_ from the atmosphere diffuses through the concrete’s pore network, dissolves in the pore water, and reacts with calcium hydroxide compounds [4]. This reduces the alkalinity of the pore water solution in the concrete cover, making it more susceptible to electrochemical corrosion, which accelerates the deterioration of concrete structures and consequently leads to a decline in concrete durability and service life [5]. According to statistical reports, the annual corrosion loss associated with buildings and infrastructure in China is approximately USD 13.985 billion, of which a major portion is attributable to reinforcement corrosion in concrete. In 2014, the total corrosion cost across all sectors in China exceeded USD 343.0 billion, accounting for approximately 3.34% of the gross domestic product (GDP) [6]. Extensive research indicates that water penetration is the primary cause of insufficient concrete durability and the susceptibility of reinforcing steel to corrosion [7,8]. Therefore, a current priority in research is the use of polymer materials to impart hydrophobic properties to concrete, thereby enhancing its resistance to water penetration and preventing the ingress of corrosive ions such as chloride and sulfate ions, thus improving the durability and corrosion resistance of concrete.

Current concrete hydrophobic modification technologies are primarily divided into two categories: polymer-based surface hydrophobic modification and internal polymer-based hydrophobic modification. Polymer-based surface hydrophobic modification involves treating the surface of cured and formed concrete to create a continuous polymer hydrophobic protective layer on the substrate surface through coating, impregnation, or formwork methods [7,9]. Xie et al. [10] treated the surface of cement substrates with a silane polymer emulsion prior to epoxy resin grouting, significantly improving the interfacial adhesion and long-term durability between the substrate and the epoxy resin. Wang et al. [11] immersed Portland cement-based specimens in an aqueous solution of fatty acid salt polymers, achieving a superhydrophobic surface after only 5 s of immersion, with a water contact angle of 154.3° and a sliding angle as low as 8.7°. Liu et al. [12] used polydimethylsiloxane (PDMS) as a negative mold to replicate lotus-leaf-like micro-nano structures on the cement surface, thereby achieving superhydrophobicity of the cement surface. Silicone polymers, fluorocarbon polymers, and acrylic polymers are widely used to modify concrete surfaces; they form a dense, water-repellent film that effectively prevents moisture penetration [4,13]. However, these surface modification methods also have many shortcomings. Environmental factors can cause concrete cracking, as well as the aging and peeling of surface coatings, leading to the degradation of the polymer surface layer. Once the hydrophobic surface is compromised, the permeability of the concrete increases dramatically because the newly exposed cement surface is hydrophilic. For example, marine structures, due to prolonged exposure to seawater, are prone to wear and spalling of the surface protective layer. This results in a decline in the concrete’s waterproofing performance and durability [14,15].

Internal hydrophobic polymer modification involves incorporating hydrophobic polymer components into fresh concrete during the mixing stage to achieve overall hydrophobization of the cement matrix [16]. Theoretically, this approach offers superior long-term durability, as even if the surface hydrophobic layer is damaged, the interior of the concrete can still maintain good hydrophobicity. Currently, the most commonly used internally-dispersed hydrophobic polymers include silane polymer emulsions, polysiloxanes, and epoxy resins, among others [17,18]. Zhang et al. [19] incorporated a silane polymer emulsion into concrete; at a dosage of 0.4%, water absorption was reduced by 70% after 28 days, but compressive strength decreased. Wu et al. [20] prepared a calcium stearate polymer emulsion using a nonionic surfactant and incorporated it into mortar, resulting in a 51.22% reduction in water absorption and a water contact angle of 130°, but compressive and flexural strengths decreased by 14.96% and 13.95%, respectively. Song et al. [2] prepared a hydrophobic mortar by blending a silane polymer emulsion with silica fume, achieving a water contact angle of 127.4° and a 59.22% reduction in water absorption coefficient, though strength decreased by 36.15%. Zhou et al. [21] modified engineering cementitious materials using a non-ionic paraffin polymer emulsion; at a 2% dosage, water absorption decreased by 58.3%, but compressive strength still decreased by 10.86%. These studies indicate that while the incorporation of hydrophobic polymers into cementitious materials significantly reduces water absorption, it also leads to a marked loss of strength. This is likely because hydrophobic substances are difficult to disperse in water and typically require the addition of emulsifiers to aid dispersion; however, the added emulsifiers can interfere with the cement hydration process, resulting in reduced concrete strength [22,23,24]. The performance of our work is compared with that of different hydrophobic systems for modifying cement-based materials reported in recent years, as shown in Table S1.

We address the issue that the aforementioned hydrophobic polymers require the addition of emulsifiers, which can affect cement hydration and lead to a decrease in strength. This study is the first to modify concrete materials using micelles prepared from self-emulsifying amphiphilic polymers as an internally incorporated hydrophobic polymer. Micelles are aggregates formed by the self-assembly of amphiphilic molecules in aqueous solution. Using allyl polyoxyethylene polyoxypropylene methyl ether and hydrogen-containing silicone oil, a silyl-hydrogen addition reaction was employed to graft polyether segments onto the siloxane backbone via stable Si-C bonds, thereby preparing water and alkali-resistant internally incorporated hydrophobic polymers. Through testing of the modified cement materials’ setting time, flowability, contact angle, water absorption, mechanical strength, and chloride ion resistance, it was verified that polyether-modified silicone oil (PMSO) micelles, when added at appropriate levels, enhance the strength of the modified cement materials and impart hydrophobicity. Additionally, changes in pore structure before and after PMSO addition were determined using low-field NMR spectroscopy. Furthermore, electrochemical accelerated-corrosion aging tests were conducted to evaluate the concrete’s corrosion resistance. The cracking time of concrete containing PMSO was significantly prolonged, indicating improved corrosion resistance.

## 2. Materials and Methods

### 2.1. Materials

Primary Reagents: Allyl polyoxyethylene polyoxypropylene methyl ether, industrial grade, average molar mass 1500 g/mol, molar ratio of ethylene oxide to propylene oxide [n(EO):n(PO)] = 1:1, Hangzhou Danwei Co., Ltd. (Hangzhou, China); hydrogenated silicone oil, industrial grade, active hydrogen mass fraction 0.18%, Zhejiang Runhe Organic Silicon New Materials Co., Ltd. (Huzhou, China); chloroplatinic acid, analytical grade, Sinopharm Chemical Reagent Co., Ltd. (Shanghai, China); and isopropyl alcohol, analytical grade, Sinopharm Chemical Reagent Co. Ltd. (Shanghai, China). Cement: P.I 42.5 Portland cement, compliant with GB 8076-2008 standard, produced by Fushun Aosai Technology Co., Ltd. (Fushun, China); fineness 0.8%, specific surface area 356 m^3^/kg, density 3.12 g/cm^3^; the mineral powder was produced by Wuhan WuXin Materials Co., Ltd. (Wuhan, China), and the fly ash was produced by Guoneng Changyuan Wuhan Qingshan Thermal Power Co., Ltd. (Wuhan, China); chemical composition is shown in Table 1. Sand: Chinese ISO standard sand, conforming to GB/T 17671 (adopted with modifications from ISO 679), produced by Xiamen Aisou Standard Sand Co., Ltd. (Xiamen, China), with a particle size range of 0.08–2 mm; the coarse aggregate was crushed limestone with a particle size of 5–20 mm. Water: Deionized water. Additives: Sodium formate, triethanolamine, diethyl mono-isopropanolamine. Water reducing agent: Polycarboxylic acid water reducing agent, designated PE900, Wuhan Subo New Materials Co., Ltd. (Wuhan, China).

### 2.2. Synthesis and Preparation of Amphiphilic Polyether-Modified Silicone Oil (PMSO) Micelles

#### 2.2.1. Synthesis of Amphiphilic Polyether-Modified Silicone Oil Polymers

**Figure 1:**
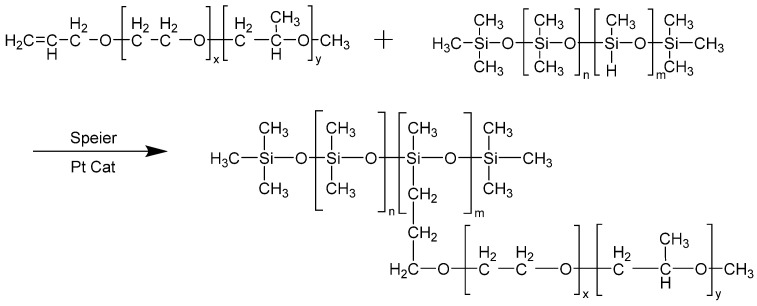
Schematic representation of the reaction equation for amphiphilic polyether-modified silicone oil polymers.

In a four-neck flask equipped with an electric stirrer, thermometer, and nitrogen protection apparatus, we sequentially added a measured amount of allyl polyoxyethylene polyoxypropylene methyl ether and a trace amount of chloroplatinic acid catalyst. Under nitrogen atmosphere, the temperature was strictly controlled at 130 °C and stirring was at 300 rpm. The reaction was conducted for 30 min. Hydrogen-containing silicone oil was slowly added dropwise via a constant-pressure separatory funnel. The stirring speed was increased to 1200 rpm while maintaining the set reaction temperature. After 8 h of reaction, polyether-modified silicone oil (PMSO) was obtained [25]. The specific synthesis reaction scheme is shown in Figure 1 below.

#### 2.2.2. Preparation of Amphiphilic Polyether-Modified Silicone Oil Micelles

**Figure 2:**
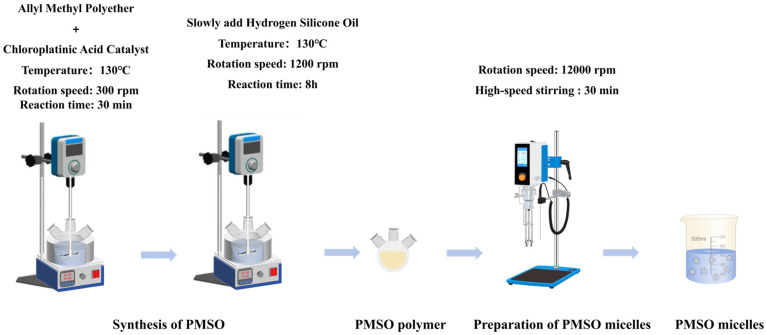
Schematic diagram of the synthesis of PMSO polymer and the preparation of PMSO micelles.

Polyether-modified silicone oil micelles were prepared via a high-speed shear homogenization method. A precisely weighed quantity of polyether-modified silicone oil was used as the self-emulsifying active component, and the required volume of deionized water was calculated based on a predetermined solid content of 20 wt%. The homogenization process was conducted under ice bath conditions using a high-speed shear homogenizer (Model HR-500 Dispersion Emulsifier, Shanghai Huxi Industrial Co., Ltd., Shanghai, China). The equipment was operated at a homogenization rotational speed of 12,000 rpm for a continuous duration of 30 min to ensure uniform micelle formation. Figure 2 illustrates the schematic diagram of the preparation of PMSO.

### 2.3. Characterization of Amphiphilic Polyether-Modified Silicone Oil Micelles

#### 2.3.1. Stability of Amphiphilic Polymers and Micelles

Storage stability evaluation and structural characterization were conducted on synthesized polyether-modified silicone oil polymers and micelles to verify the integrity of their molecular structures during long-term storage. The polymer and micelle samples were sealed separately in glass reagent bottles and stored at room temperature for three months.

#### 2.3.2. Structural Characterization of Polyether-Modified Silicone Oil Polymers

The synthesized polyether-modified silicone oil was characterized using a Nicolet 6700 Fourier Transform Infrared Spectrometer (Waltham, MA, USA) within the wavelength range of 4000–400 cm^−1^, with a scan step of 1 cm^−1^. A small amount of emulsifier was dissolved in deuterated chloroform and subjected to ^1^H NMR testing using a Bruker 400 MHz liquid nuclear magnetic resonance (NMR) spectrometer (Rheinstetten, Germany).

#### 2.3.3. Determination of HLB Value of Polyether-Modified Silicone Oil Polymers

Following H.L. Greenward’s modified titration method, 0.2 g of the sample was dissolved in 20 mL of dioxane/benzene (90 vol% and 4 vol%). It was titrated with deionized water until a distinct turbidity became visible. The volume of deionized water used (V) was recorded in milliliters and the HLB value calculated using the following formula.

(1) $$\mathrm{HLB}=23.641\;\log_{10}V-10.16$$

#### 2.3.4. Gel Permeation Chromatography (GPC) of Polyether-Modified Silicone Oil Polymers

The molecular weight and molecular weight distribution of the samples were determined using an Agilent 1260 Infinity II system (Waldbronn, Germany). The temperature was set at 40 °C, tetrahydrofuran (THF) was used as the mobile phase, and the flow rate was 1.0 mL/min.

#### 2.3.5. Characterization of Micelle Size of Polyether-Modified Silicone Oil

After diluting the micellar solution 20-fold, the particle size was measured by dynamic light scattering (DLS) using a Malvern Zetasizer Nano ZS90 (Malvern, UK).

### 2.4. Preparation of Cement Specimens

Cement paste was prepared with a water–cement ratio of 0.29. The mortar water–cement ratio was 0.44, with a sand-to-cement mass ratio of 3:1. The concrete water–binder ratio was 0.3588. PMSO micelles were added to cement at concentrations of 0%, 0.1%, 0.2%, 0.3%, 0.4%, and 0.5% by cement mass (designated as Control, 1P, 2P, 3P, 4P, and 5P, respectively). PMSO micelles were added to concrete at 0%, 0.1%, 0.2%, 0.3%, 0.4%, and 0.5% by mass of cementitious materials (designated as Control, 1CP, 2CP, 3CP, 4CP, and 5CP, respectively). PMSO micelles had to be pre-mixed with water for 5 min to ensure uniform dispersion without bubbles. The mixing water volume was adjusted based on micelle content to account for micelle moisture content. The cement paste formulation is detailed in Table 2, the cement mortar composition ratios in Table 3, and the concrete composition ratios in Table 4.

Note: Water refers to the total water content in the design mix, including polycarboxylic acid superplasticizer and additives such as sodium formate, triethanolamine, diethyl mono-isopropanolamine (in a 2:1:1 ratio), and PMSO micelles. P900 is the code for Subo Company’s polycarboxylic acid superplasticizer concentrate. The direct addition rate is 2%, equivalent to 8.5 kg/m^3^. The direct addition rate for additives is 2%.

### 2.5. Liquidity Testing

According to national standard GB/T 2419-2005, the flowability of cement mortar specimens with different cement content was determined using the NLD-3 flow meter (Wuxi, China). After the jump test, the diameter of the mortar base was measured in two mutually perpendicular directions using calipers. The average of three tests was taken as the final test result.

### 2.6. Standard Consistency Water Content (SCWC) and Setting Time

According to GB/T 1346-2011, this study employed a Vicat apparatus to determine the SCWC and setting time of cement paste containing different content of PMSO. During testing, the test rod freely sinks vertically into the cement paste under gravity. When the rod settles to a distance of 5–7 mm from the base plate, the paste is deemed to have reached standard consistency. The percentage of mixing water relative to the cement mass at this point constitutes the SCWC value. For setting time determination, the initial setting time was defined as the duration required for the test needle to sink to 3–5 mm above the base plate; final setting time was determined when the needle sank 0.5 mm into the paste with no residual marks on the ring attachment. All data were obtained by calculating the arithmetic mean from three independent parallel tests to ensure reproducibility and reliability of results.

### 2.7. Fourier Transform Infrared (FTIR) and X-Ray Diffraction (XRD) Testing

Chemical functional groups in mortar sample powders were analyzed using Nicolet 6700 Fourier Transform Infrared Spectrometer (Waltham, MA USA). The scanning range was 4000–400 cm^−1^. The hydration products of pure mortar samples at different stages were analyzed using the X-ray diffractometer (XRD), Rigaku SmartLab SE, Rigaku Corporation (Tokyo, Japan). Prior to analysis, samples were ground into powder and dried in an oven. Measurements were performed using Cu Kα radiation (λ = 0.154056 nm) within the 2θ range of 5–90°.

### 2.8. Scanning Electron Microscopy (SEM) Testing

The microstructure of the pure paste samples cured for 28 days was observed using a field emission scanning electron microscope (FESEM, FEI, QUANTA F250, Hillsboro, OR, USA).

### 2.9. Water Contact Angle Measurement

After curing mortar samples for 28 days, they were cut into small pieces using a cutting machine for water contact angle measurement. The cut surfaces of the samples received no special treatment. Following cutting, the samples were rinsed with deionized water and dried in a vacuum drying oven. Water contact angles on both the inner and outer surfaces of the samples were measured using an optical contact angle meter (JC2000C, Powerach, Shanghai, China). A 6 μL droplet was placed at four distinct positions on the sample surface to measure the contact angle. The average of the four measurements served as the test result for the water contact angle.

### 2.10. Water Absorption Measurement

According to Chinese Standard JC474-2008, we determined the water absorption rate of mortar samples (dimensions 70.7 × 70.7 × 70.7 mm^3^) and concrete samples (dimensions 100 × 100 × 100 mm^3^) after 28 days. First, after curing the mortar and concrete specimens for 28 days, they were dried in an oven at 75–80 °C until constant weight was achieved. Subsequently, the specimens were placed in a tank with two reinforcing bars at the bottom. The specimens were submerged to a depth of 35 mm, with the water level maintained constant. The weight changes of the specimens were measured at different time intervals. The test result is the average value of the three samples.

### 2.11. Porosity Measurement

The low-field NMR analyzer (NMIConsole) from Suzhou Nuomai Analytical Instruments Co., Ltd., Suzhou, China, was used to determine the porosity and pore size distribution of mortar samples (cylinders with a diameter and height of 2 cm) after 28 days. The test result is the average value of the three samples.

### 2.12. Mechanical Strength Measurement

The flexural and compressive strengths of mortar were tested using a flexural testing machine and a compressive testing machine, respectively, in accordance with GB/T 17671-1999 “Test method for strength of cement mortar (ISO method)”. Prismatic specimens with dimensions of 40 mm × 40 mm × 160 mm were cured for 3, 7, and 28 days before testing. The flexural strength was determined using a three-point loading method with a span of 100 mm and a loading rate of 50 N/s. Three specimens were tested for each curing age, and the reported flexural strength was the average of the three measurements, accurate to 0.1 MPa. After the flexural test, each specimen fractured into two half prisms, each approximately 40 mm × 40 mm × 80 mm in size. The compressive strength was then measured on the molded side faces (40 mm × 40 mm) of the half prisms at a loading rate of 2400 N/s. For each curing age, a total of six half prisms (obtained from three complete prisms) were tested, and the compressive strength was reported as the arithmetic mean of the six values, accurate to 0.1 MPa.

### 2.13. Chloride Ion Resistance Test

In accordance with the national standard GB/T 50082-2009, the rapid chloride migration method (RCM) was employed to determine the chloride ion resistance of concrete specimens after 56 days of curing. First, a 0.3 mol/L sodium hydroxide solution was prepared 24 h in advance as the anode solution, and a 10 wt% sodium chloride solution was prepared as the cathode solution. Vacuum-treated concrete specimens (100 mm diameter, 50 mm height) were placed into rubber sleeves, which were then inserted into test vessels maintained at 20–25 °C. Test duration and applied voltage were determined by the initial current generated at 30 V. After testing, specimens were bisected diametrically, and the cut surfaces were immediately sprayed with 0.1 mol/L silver nitrate solution. The test results are the average of three specimens and are expressed as the mean ± standard deviation.

The formula for calculating the chloride ion migration coefficient (D_RCM_) is as follows:

(2) $$D_{\mathrm{RCM}}\;=\;\frac{0.0239\;\times \;(273\;+\;T)L}{(U-2)t}(X_{d}-0.0238\sqrt{\frac{(273\;+\;T)LX_{d}}{U-2}})$$

In the equation, D_RCM_ denotes the non-steady-state chloride ion migration coefficient of concrete (m^2^/s), accurate to 0.1 × 10^−12^ m^2^/s; U represents the absolute value of the applied voltage (V); T is the average of the initial and final temperatures of the anode solution (°C); L indicates the specimen thickness (mm); X_d_ signifies the chloride ion penetration depth (mm); and t denotes the test duration (h).

### 2.14. Concrete Corrosion Resistance Testing

#### 2.14.1. Sample Preparation

The specimens for the corrosion resistance test were concrete cylinders measuring 150 mm in height and 100 mm in diameter. A steel bar, electrically connected by copper wire at one end (100 mm diameter × 150 mm length), was centrally embedded within each cylinder. The steel bar was embedded in the concrete cylinder with its bottom positioned at least 50 mm from the cylinder’s base. To eliminate crevice corrosion between the steel bar and concrete, the concrete cylinder’s exposed surface was coated with epoxy resin. Prior to casting the specimen, the steel bar’s surface was cleaned with a wire brush to remove surface rust. The specimen was placed in the casting chamber for 24 h, then demolded and cured to the test age.

#### 2.14.2. Accelerated Corrosion Test

**Figure 3:**
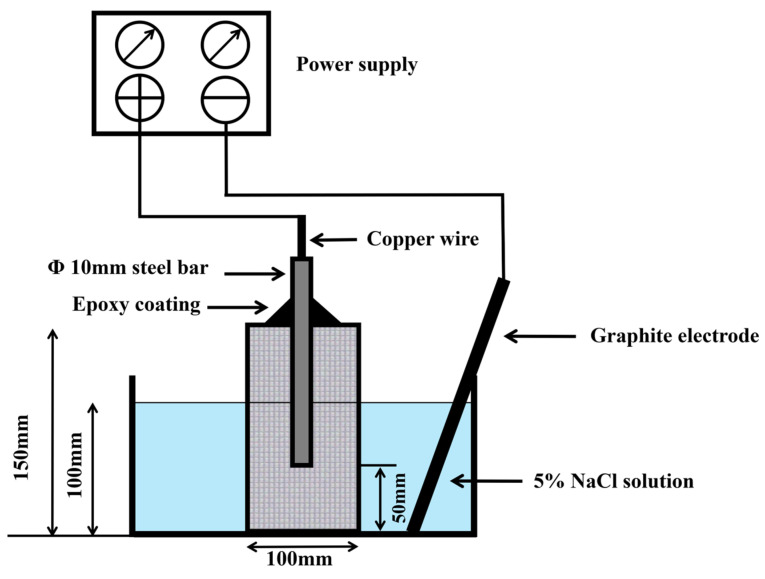
Schematic diagram of the accelerated-corrosion test apparatus.

The corrosion resistance of concrete specimens can be determined through accelerated corrosion testing. Testing is conducted at the 56-day age of the concrete. Concrete cylinders embedded with 10 mm diameter steel bars are immersed in a 5% NaCl solution by weight of water. The corrosion process is initiated by applying a 30 V anodic potential to the specimens, using a steel rod as the positive electrode, a stone rod as the negative electrode, and the sodium chloride solution as the electrolyte. An external constant voltage is applied to accelerate corrosion and shorten the test cycle, adapting to practical laboratory testing conditions. Figure 3 shows a schematic diagram of the experimental setup for the accelerated corrosion test.

#### 2.14.3. Electrochemical Measurements

The CHI600E electrochemical workstation was used to test specimens at different intervals (0 h, 48 h, 168 h, and the time of test cracking) during accelerated corrosion. The corrosion state was evaluated using dynamic potential scanning (Tafel curves). The electrochemical tests employed a graphite rod as the counter electrode, a saturated calomel electrode (SCE) as the reference electrode, and the steel reinforcement within the specimen as the working electrode. Tafel plots were recorded between −1 V and +1 V at a scan rate of 2 mV/s.

## 3. Results and Discussion

### 3.1. Synthesis and Characterization of Amphiphilic Polyether-Modified Silicone Oil (PMSO)

#### 3.1.1. Synthesis of Amphiphilic PMSO

Amphiphilic polyether-modified silicone oil is an organosilicon polymer with a hydrophobic siloxane backbone and incorporated hydrophilic polyether groups [26,27,28]. The polyether groups possess good hydrophilicity, which enhances dispersion in water and allows them to interact with water molecules to form stable micelles, while the hydrophobic siloxane backbone provides the concrete with overall hydrophobic properties. Since concrete is a strongly alkaline environment, the hydrophobic polymers prepared must possess properties such as water resistance, alkali resistance, and weather resistance. Therefore, amphiphilic polyether-modified silicone oil (PMSO) was synthesized via a silyl-hydrogen addition reaction using allyl polyoxyethylene-polyoxypropylene methyl ether and hydrogen-containing silicone oil [25]. In this reaction, polyether segments are grafted onto the siloxane backbone via stable Si–C bonds. The siloxane backbone consists of hydrolysis-resistant Si–O–Si bonds, thereby avoiding the drawback of unstable, weak bonds such as Si–C–O and Si–O–C, which are prone to hydrolysis under alkaline conditions.

Hydrophobic polymer-modified concrete must exhibit both hydrophobicity and a certain degree of dispersibility. The ratio of hydrophobic to hydrophilic components significantly affects the properties of the final product. Therefore, a series of hydrophobic polymers with different HLB values (denoted as HLB_th_) were synthesized by varying the molar ratio of polyether to silicone oil. The experimental results are shown in Table 5. When the HLB_th_ value is 3, the product appears cloudy, primarily due to an insufficient amount of polyether and an incomplete reaction; as the HLB_th_ value increases, the product forms a clear, uniform, and stable system. The gel permeation chromatography (GPC) results of polymers with different HLB values are presented in Table 5. The relatively high polydispersity is attributed to the presence of a small amount of low-molecular-weight raw materials in the polymer system.

#### 3.1.2. Measured HLB Values of PMSO

Following H.L. Greenward’s modified titration method, the volume of water required to cause cloudiness was recorded, and the measured HLB value (denoted as HLB_exp_) was calculated using the formula. The results are shown in Table 6 below. As can be seen from the table, the measured HLB values are all higher than the designed HLB values, with an error of approximately 8%. This may be due to a small amount of unreacted hydrophilic polyether raw material remaining in the system, which slightly increased the overall hydrophilicity of the product. However, since the difference between the measured HLB values and the designed HLB values is not significant, this indicates that the reaction was completed to a high degree.

#### 3.1.3. FTIR Characterization

**Figure 4:**
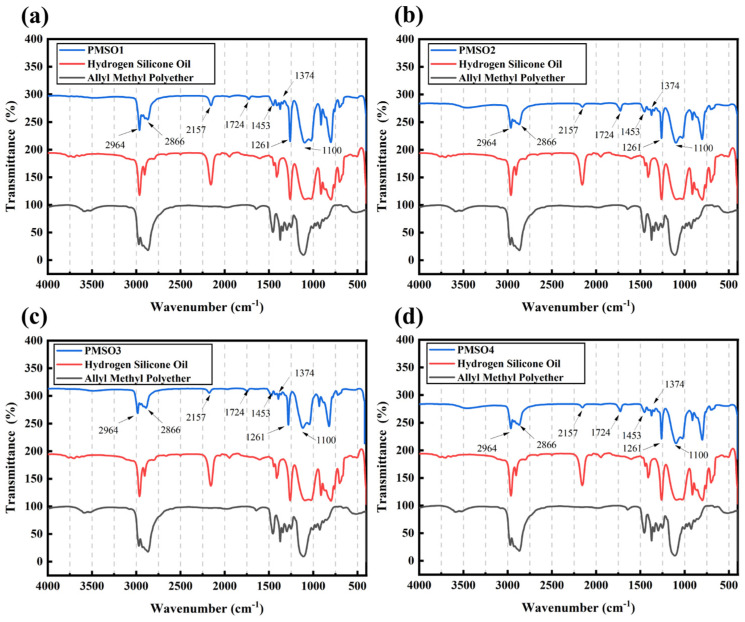
Infrared spectra of polyether-modified silicone oils with different HLB values: (**a**) PMSO1; (**b**) PMSO2; (**c**) PMSO3; and (**d**) PMSO4.

The infrared spectra of polyether-modified silicone oil polymers synthesized with different HLB values are shown in Figure 4. The characteristic absorption peaks at 2964 cm^−1^ and 2866 cm^−1^ correspond to the stretching vibrations of –CH_3_ and –CH_2_ groups, respectively; the characteristic absorption peaks at 1453 cm^−1^ and 1374 cm^−1^ correspond to the bending vibrations of –CH_3_ and –CH_2_ bending vibrations, while the peak at 1261 cm^−1^ corresponds to the characteristic peak of the Si–CH_3_ bond, and the peak between 1100–1027 cm^−1^ represents an overlap of the Si–O–Si and C–O–C peaks [25]. The characteristic absorption peak at 2157 cm^−1^ in the hydrogen-containing silicone oil spectrum corresponds to the stretching vibration of the silane hydrogen bond. The significant weakening of the characteristic absorption peak at the same position in the synthetic product indicates that the silane hydrogen bond participated in the reaction. As the HLB value increases, the intensity of the characteristic absorption peak at 2157 cm^−1^ corresponding to the stretching vibration of the silane hydrogen bond gradually weakens. This is due to the rise in HLB value, which results in an increased amount of polyether; consequently, more polyether segments react with the silane hydrogen bonds in the hydrogen-containing silicone oil via silane hydrogen addition reactions. As the reaction proceeds, a large number of silane hydrogen bonds are consumed. Compared to the spectrum of hydrogen-containing silicone oil, the peaks at 2964–2869 cm^−1^ and 1095–1020 cm^−1^ have broadened and intensified. The –CH_3_, –CH_2_, and C–O–C groups in the polyether contribute to the absorption enhancement at these two regions, indicating that the reaction between the polyether and the hydrogen-containing silicone oil is essentially complete.

#### 3.1.4. ^1^H NMR Characterization

**Figure 5:**
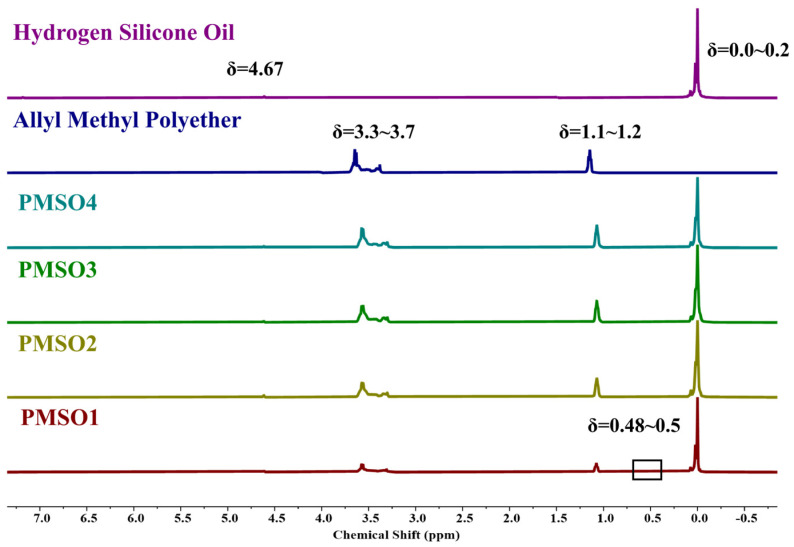
NMR hydrogen spectra of polyether-modified silicone oil synthesis products with different HLB values.

The NMR hydrogen spectra of polyether-modified silicone oils with different HLB values are shown in Figure 5. In hydrogen-containing silicone oil, the methyl hydrogen peak directly linked to silicon appears near a chemical shift of δ = 0~0.2, while the peak at δ = 4.67 corresponds to the siloxane bond in hydrogen-containing silicone oil. The proton peak of the polyether raw material near δ = 1.1~1.2 corresponds to the methyl hydrogen peak in the polyoxypropylene segment of the polyether, the absorption peak of the terminal methyl proton of the polyether appears near δ = 3.37, the proton absorption peaks of the methylene and methyl hydrogen in the polyether chain segments are located at δ = 3.3~3.7, and the solvent peak of CDCl_3_ is at δ = 7.26. In the spectrum of the product, polyether-modified silicone oil, the intensity of the Si-H proton peak at δ = 4.67 is significantly reduced, while a new methylene peak linked to silicon appears at δ = 0.48~0.5. Furthermore, the proton peaks of the polyether chain segments coexist with those of the silanol groups, indicating that the hydrogen-containing silicone oil and allyl polyether have successfully grafted via a silyl-hydrogen addition reaction. The peak corresponding to the hydrogen on the carbon–carbon double bond in the polyether has almost disappeared, and the ratio of the area under the curve of the silyl-hydrogen bond peak to that of the methyl peak in the hydrogen-containing silicone oil has significantly decreased, indicating that the silyl-hydrogen addition reaction is nearly complete. Combined with the analysis of infrared spectroscopy data, it can be confirmed that the hydrogen-containing silicone oil and the allyl polyoxyethylene-polyoxypropylene methyl ether in the system have completed the silyl-hydrogen addition reaction, resulting in the synthesis of the target product, polyether-modified silicone oil.

The conversion rate of the silanol groups was calculated based on the NMR peak areas of the polyether-modified silicone oil mixtures with different HLB values. The results are shown in Table 7. The actual conversion rates of the silanol groups were all higher than the theoretical values, possibly due to self-crosslinking reactions involving the silanol groups and the fact that the hydrogen-containing silicone oil was in excess in the reaction system, resulting in slightly higher conversion rates [29].

### 3.2. Characterization of Polyether-Modified Silicone Oil (PMSO) Micelles

Given that cement pore solution is characterized by high alkalinity and high ionic strength, the stability of PMSO micelles was systematically evaluated to investigate the mechanism underlying their hydrophobic modification effect on cement-based materials and to verify their applicability in the cement pore solution environment.

#### 3.2.1. Characterization of the Particle Size of PMSO Micelles

**Figure 6:**
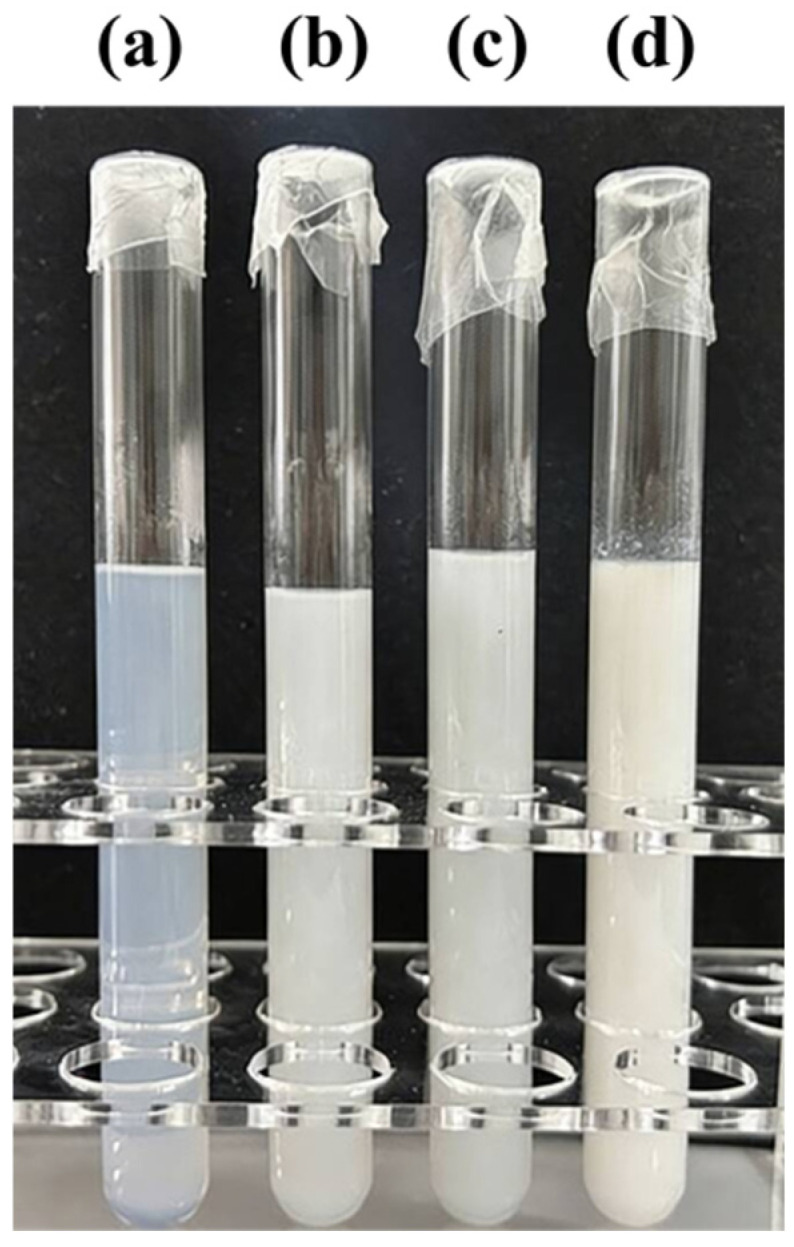
Schematic of micelles formed by polyether-modified silicone oil with different HLB values: (**a**) PMSO4; (**b**) PMSO3; (**c**) PMSO2; and (**d**) PMSO1.

Because polyether-modified silicone oil is hydrophilic, it possesses good self-emulsifying properties and can self-emulsify in water to form a stable micellar structure, as shown in Figure 6 below. Particle size analysis was performed on micelles of polyether-modified silicone oil with different HLB values, and the results are shown in Table 8 below. As shown in the table, the self-emulsifying properties indicate that as the HLB value increases from 3.5 to 5, the appearance of the polyether-modified silicone oil micelles gradually changed from a white liquid to a pale white liquid, and finally to a pale blue transparent liquid, while the average particle size decreased from 161.4 nm to 79.9 nm. This indicates that an increase in the HLB value enhances the hydrophilicity of the polymer, thereby facilitating its more thorough dispersion in water and resulting in more uniform micelles with smaller particle sizes.

#### 3.2.2. Characterization of the Critical Micelle Concentration (CMC) of PMSO Micelles

**Figure 7:**
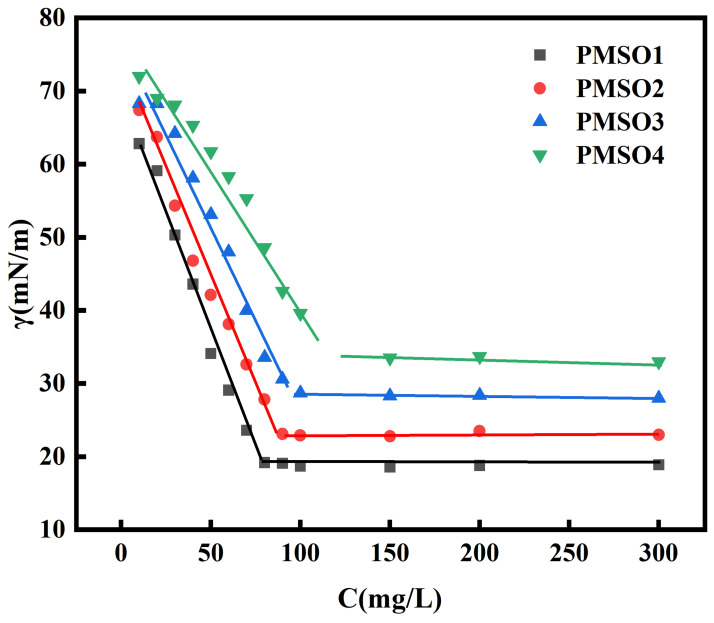
Relationship between surface tension and concentration of PMSO micelles with different HLB values.

The critical micelle concentration (CMC) of PMSO micelles was determined as shown in Figure 7 below. For PMSO micelles with HLB values ranging from 3.5 to 5, the minimum surface tension values were 18.94 mN/m, 23.06 mN/m, 28.65 mN/m, and 33.80 mN/m, and the corresponding CMC values were 77.30 mg/L, 87.09 mg/L, 95.08 mg/L, and 115.08 mg/L, respectively. The results indicate that as the HLB value increases, the CMC increases from 77.30 mg/L to 115.08 mg/L, and the surface tension increases from 18.94 mN/m to 33.80 mN/m. Both parameters are positively correlated with the HLB value. The higher the HLB, the stronger the hydrophilicity, and the higher the concentration required for micelle formation, leading to an increase in CMC.

#### 3.2.3. Characterization of the Stability of PMSO Micelles

**Figure 8:**
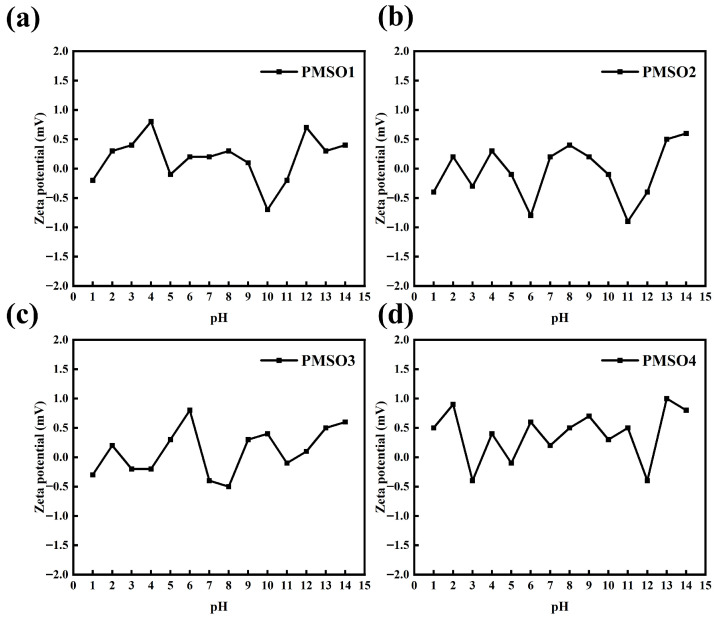
Variation of Zeta potential with pH for micelles with different HLB values: (**a**) PMSO1; (**b**) PMSO2; (**c**) PMSO3; and (**d**) PMSO4.

**Figure 9:**
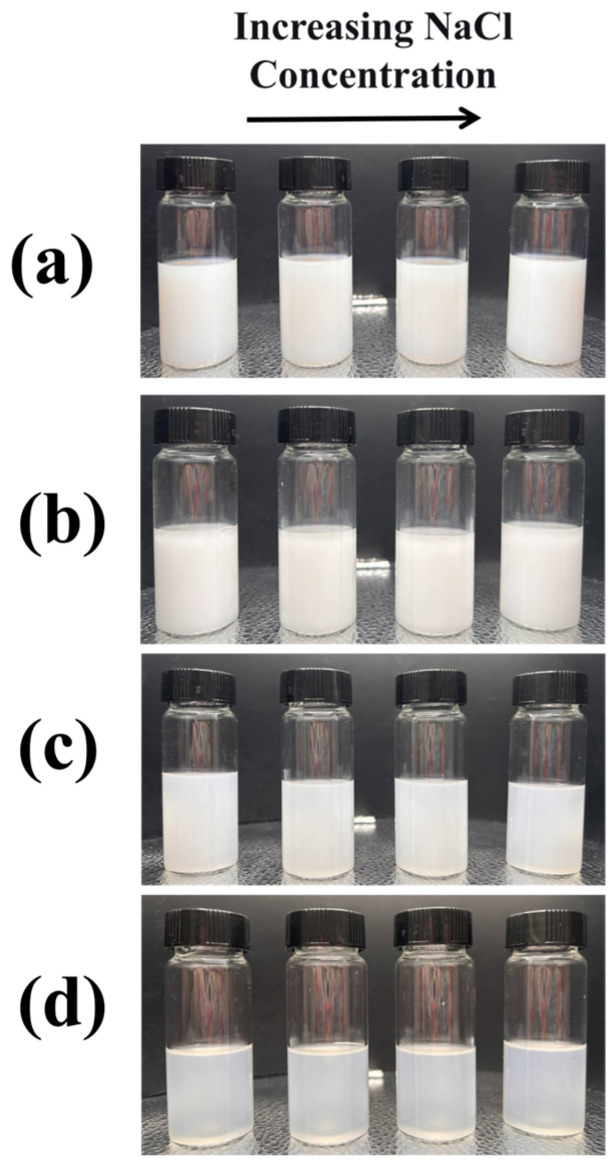
Stability of different PMSO micelles in NaCl solutions with increasing concentration: (**a**) PMSO1; (**b**) PMSO2; (**c**) PMSO3; and (**d**) PMSO4.

The variation of Zeta potential with pH for PMSO micelles with different HLB values is shown in Figure 8. The experimental results demonstrate that the Zeta potential of PMSO micelles remains between −1 and 1 mV across different pH values, with no significant change over the entire pH range. Since PMSO is a nonionic micelle and its surface contains no ionizable groups, the Zeta potential is close to zero and does not vary with pH [30,31]. The stability of micelles with different HLB values under high ionic strength was evaluated. NaCl solutions at concentrations of 0.1, 0.2, 0.5, and 1.0 mol/L were added to the micelle dispersions, and the stability was examined. After standing, no phase separation or precipitation was observed for any of the samples, indicating that the PMSO micelles possess good stability under high ionic strength conditions. Furthermore, dynamic light scattering (DLS) was employed to measure the particle size. The results showed that the particle size remained essentially unchanged in NaCl solutions of different concentrations. The specific results are shown in Figure 9 and Table 9 below.

All prepared micelles remained free of phase separation or precipitation after three months of storage at room temperature. This indicates that micelles prepared with different HLB values possess excellent storage stability, meeting the requirements for incorporation into cement-based materials. They can serve as hydrophobic polymers for hydrophobic modification of cement-based materials.

### 3.3. Effect of Polyether-Modified Silicone Oil Micelles with Different HLB Values on the Water Absorption of Mortar

**Figure 10:**
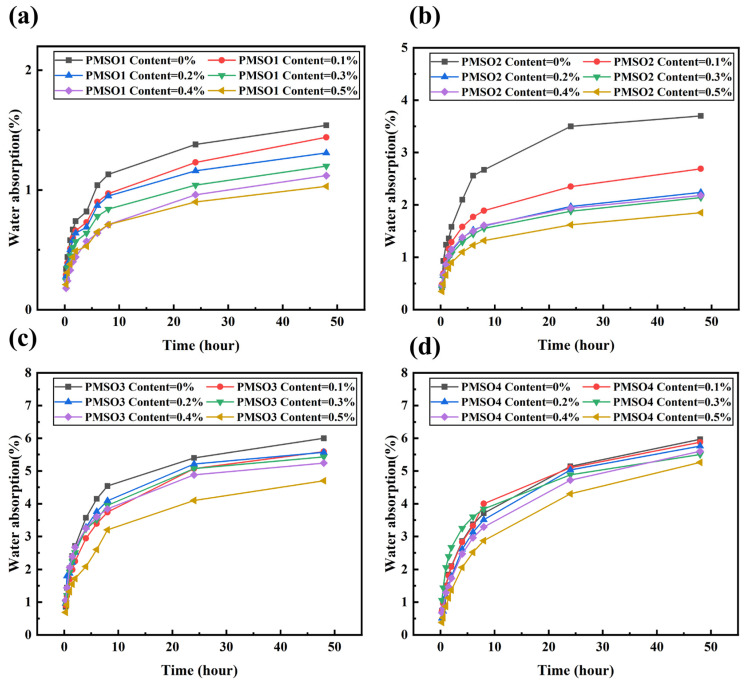
Water absorption of mortar specimens with different PMSO content: (**a**) PMSO1; (**b**) PMSO2; (**c**) PMSO3; and (**d**) PMSO4.

The effect of modifying cement mortar with polyether-modified silicone oil micelles of different HLB values at various content levels on water absorption was investigated. The experimental results are shown in Figure 10. As the content of polyether-modified silicone oil micelles increased, the water absorption of the mortar specimens gradually decreased compared to the control group, and the rate of decrease slowed over time. Specifically, the blank group and the 0.5 wt% PMSO1-modified group exhibited water absorption rates of 1.54% and 1.03%, respectively, after 48 h, representing a 33.12% reduction in water absorption. For PMSO2, the water absorption rates of the blank group and the 0.5 wt%-doped group at 48 h were 3.7% and 1.84%, respectively, representing a 50.27% reduction. For PMSO3, the water absorption rates of the blank group and the 0.5 wt%-doped group at 48 h were 6% and 4.7%, respectively, representing a 21.67% reduction. For PMSO4, the water absorption rates of the blank group and the 0.5 wt% addition group at 48 h were 5.97% and 5.26%, respectively, representing a reduction of 11.89%. The extent to which PMSO polymers reduce the water absorption rate of cement mortar exhibits a trend of first increasing and then decreasing as the HLB value rises. At an HLB value of 4, the best hydrophobic effect is observed, with the most significant reduction in water absorption rate. When the HLB value is too high, the increased number of hydrophilic segments in the polymer leads to excessive hydrophilicity, reducing hydrophobic efficiency and significantly weakening the improvement in water absorption rate.

### 3.4. Effect of Polyether-Modified Silicone Oil Micelles with Different HLB Values on the Mechanical Properties of Mortar

Mechanical strength is the most critical criterion for evaluating cement-based concrete materials; its level directly determines a structure’s load-bearing capacity, durability, and service life. When incorporating hydrophobic polymers such as polyether-modified silicone oil, the goal is not only to achieve hydrophobicity but also to ensure that the material possesses good mechanical properties, thereby preventing a decline in strength due to delayed hydration.

**Figure 11:**
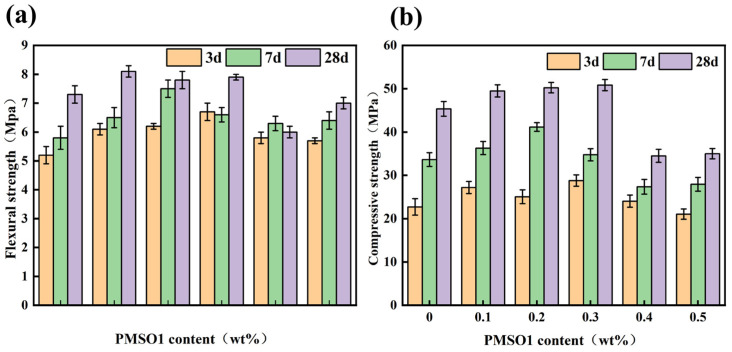
The effect of different content of PMSO1 on the mechanical strength of modified cement mortar: (**a**) flexural strength; (**b**) compressive strength.

The effect of different PMSO1 content on the mechanical strength of modified cement mortar is shown in Figure 11. At low PMSO1 content, both flexural strength and compressive strength increased. The 28-days flexural strength of cement mortar containing 0.1 wt% PMSO1 was 10.96% higher than that of the control group, and the compressive strength was 9.15% higher. However, as the content increased, both flexural and compressive strengths decreased at high levels. For cement mortar with a PMSO1 content of 0.5 wt%, the 28-days flexural strength was 4.11% lower than that of the control group, and the compressive strength was 22.82% lower. This indicates that when PMSO1 is added to cement mortar, low content has a positive effect on the mortar’s strength, whereas high content reduces its strength.

**Figure 12:**
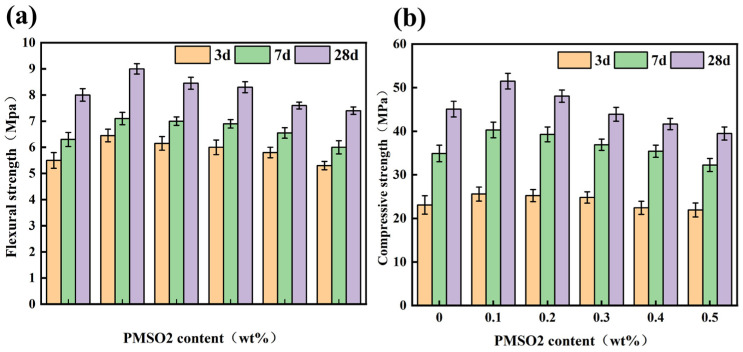
The effect of different content of PMSO2 on the mechanical strength of modified cement mortar: (**a**) flexural strength; (**b**) compressive strength.

The effect of different PMSO2 content on the mechanical strength of modified cement mortar is shown in Figure 12. At a PMSO2 content of 0.1 wt%, the 28-days flexural strength of the cement mortar increased by 12.5% compared to the control group, and the compressive strength increased by 14.19%. This is because PMSO possesses a certain degree of surface activity, which reduces interfacial tension. Consequently, during the cement hydration process, it adsorbs onto the surface of cement particles, enhancing their stability, resulting in smaller particles of hydration products and increased density. However, as the admixture content increases, the compressive strength decreases. During the early stages of cement hydration, the adsorption of PMSO micelles slightly affects water flow and diffusion. Consequently, the hydration and gelation rates are slower compared to the control group, which influences the crystal structure of the hydration products. However, this has little effect on the degree of hydration. The addition of PMSO allows for complete hydration in the later stages, as evidenced by the 28-days strength being consistent with or slightly higher than that of the control group.

**Figure 13:**
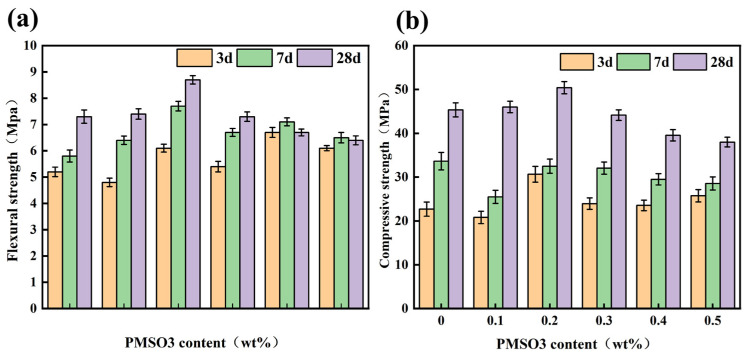
The effect of different content of PMSO3 on the mechanical strength of modified cement mortar: (**a**) flexural strength; (**b**) compressive strength.

The effect of different PMSO3 content on the mechanical strength of modified cement mortar is shown in Figure 13. When the PMSO3 content was 0.1 wt%, the compressive strength was lower than that of the control group in the early stages, but it increased significantly by 28 days and exceeded that of the control group. When the PMSO3 content was 0.5 wt%, both the early-stage compressive and flexural strengths were higher than those of the control group, but the later-stage strengths were lower than those of the control group.

**Figure 14:**
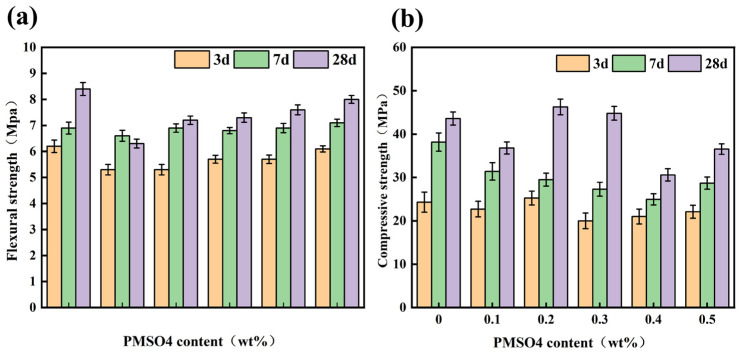
The effect of different content of PMSO4 on the mechanical strength of modified cement mortar: (**a**) flexural strength; (**b**) compressive strength.

The effect of different PMSO4 content levels on the mechanical strength of modified cement mortar is shown in Figure 14. The addition of PMSO4 reduced both the flexural and compressive strengths of the cement mortar, indicating that PMSO4 has a significant negative impact on the mechanical properties of the cement mortar.

### 3.5. Effects of Different PMSO2 Content on Cement Paste and Mortar

A comprehensive comparison of the effects of polyether-modified silicone oil on the mechanical properties and water absorption of cement mortar indicates that PMSO2 can significantly improve the flexural and compressive strengths of the mortar even at low dosages, has minimal negative impact on early-stage strength, and exhibits outstanding hydrophobic properties with the greatest reduction in water absorption. Based on a comprehensive evaluation of mechanical properties, hydrophobic performance, and system stability, the PMSO2 system was selected as the optimal formulation for further research.

#### 3.5.1. Effects of Standard Consistency Water Content (SCWC) and Flowability

**Figure 15:**
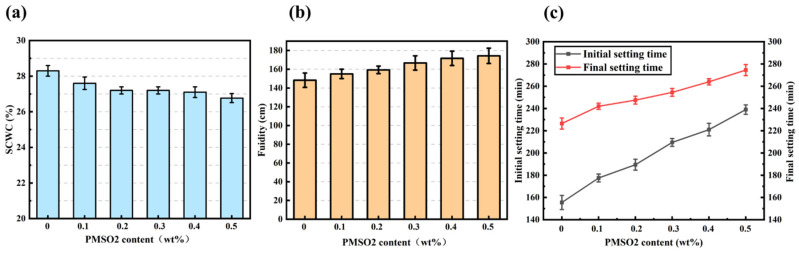
(**a**) Effect of PMSO2 content on the SCWC of cement paste; (**b**) effect of PMSO2 content on the flowability of cement mortar; and (**c**) initial and final setting times of PMSO2-modified cement paste.

The effect of different PMSO2 content on the specific water requirement (SCWC) of cement paste was investigated. As shown in Figure 15a, the modified cement paste required less water than the unmodified cement paste. The higher the PMSO content, the lower the water requirement of the PMSO2-modified cement paste. This indicates that PMSO2 micelles act as lubricants in the cement paste, thereby reducing its SCWC. As the PMSO2 content increased, the water requirement of the cement paste decreased, indicating that the lubricating effect was enhanced with increasing PMSO2 content. The effect of PMSO2 content on cement mortar fluidity is shown in Figure 15b. The results indicate that the fluidity of PMSO2-modified cement mortar is consistently higher than that of the control group. Furthermore, as the PMSO2 content increases, the fluidity of the cement mortar also increases. This demonstrates that PMSO2 has a significant dispersing effect on cement particles. The hydrophilic polyethylene oxide segments in the polyether moiety adsorb onto cement particle surfaces via hydrogen bonding with surface hydroxyl groups, while the hydrophobicity of Si-O bonds in hydrogen-containing silicone oil reduces cement particle surface tension, thereby enhancing mortar fluidity [32]. As shown in Figure 15c, both the initial setting time and final setting time of PMSO2-modified cement paste increase with rising PMSO2 content.

#### 3.5.2. FTIR and XRD Analysis

**Figure 16:**
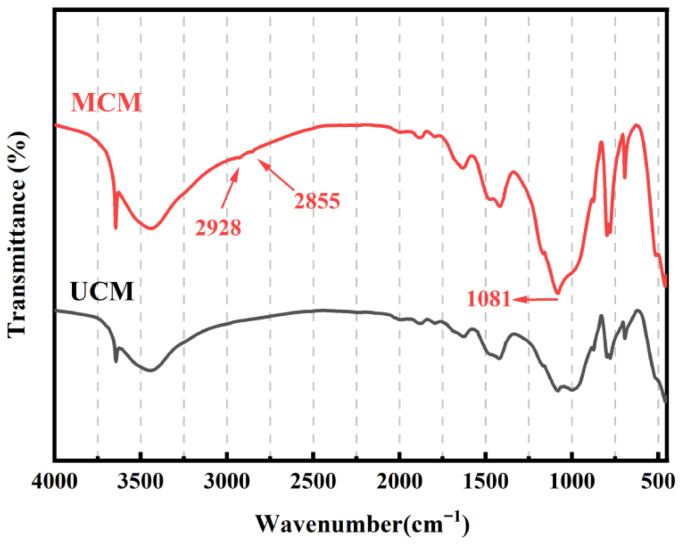
Infrared characteristic spectra of unmodified cement mortar (UCM) and modified cement mortar (MCM) with 0.5 wt% PMSO2 content.

Figure 16 shows the infrared characteristic spectra of unmodified cement mortar (UCM) and modified cement mortar (MCM) with a PMSO2 content of 0.5 wt%. Noticeable differences in absorption peaks at certain wavelengths are observed between the unmodified and modified cement mortars. The peaks at 2928 cm^−1^ and 2855 cm^−1^ correspond to the asymmetric stretching vibration of –CH_3_ and the symmetric stretching vibration of –CH_2_ in the polyether segment of PMSO2, respectively [33]. These peaks are consistent with the FTIR characterization results of pure PMSO2. These absorption peaks are absent in the unmodified sample. In the 1000–1100 cm^−1^ region, the peak of the cement hydration product calcium silicate hydrate (C–S–H) gel overlaps with the Si–O–Si peak of PMSO2 micelles. The absorption peak at 1081 cm^−1^ exhibits a noticeably sharper peak shape and higher intensity. The infrared spectral analysis results indicate that PMSO2 is uniformly distributed throughout the cement mortar.

**Figure 17:**
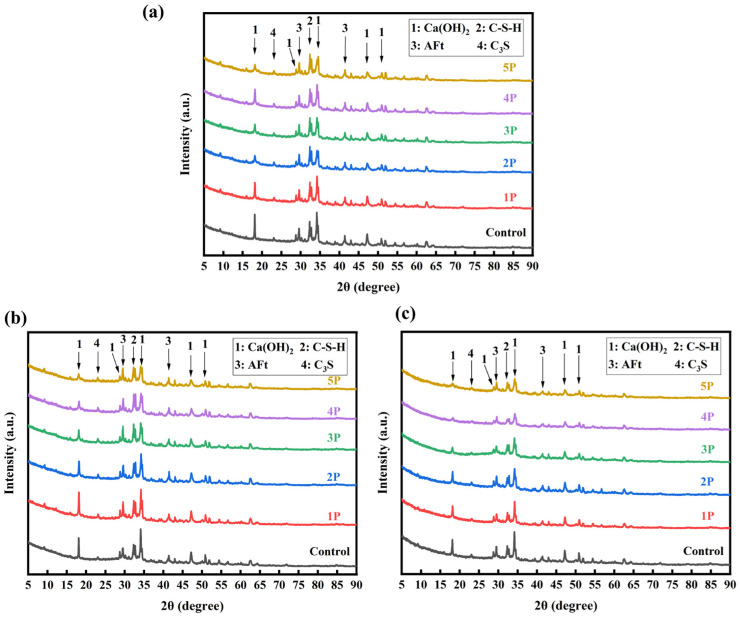
(**a**–**c**): XRD patterns of hydration products corresponding to cement paste at 3d, 7d, and 28d for PMSO2 content of 0%, 0.1 wt%, 0.2 wt%, 0.3 wt%, 0.4 wt%, and 0.5 wt%.

Figure 17a–c show the XRD patterns of hydration products corresponding to 3d, 7d, and 28d for cement paste with PMSO2 content of 0%, 0.1 wt%, 0.2 wt%, 0.3 wt%, 0.4 wt%, and 0.5 wt%, respectively. The primary cement hydration products are calcium hydroxide [Ca(OH)_2_] and ettringite (AFt). Incorporating PMSO2 did not alter the types of hydration products but influenced their quantities [34]. It can be observed that as the amount of polyether-modified silicone oil micelles increases, the intensity of the calcium hydroxide diffraction peak decreases. This may be due to the addition of PMSO2 affecting the crystal structure of the hydration products compared to the control group, limiting the natural growth of crystals and resulting in an incomplete structure, thereby causing a decrease in peak intensity. Macroscopically, this manifests as an extension of the initial and final setting times.

#### 3.5.3. Surface Microstructure Analysis

**Figure 18:**
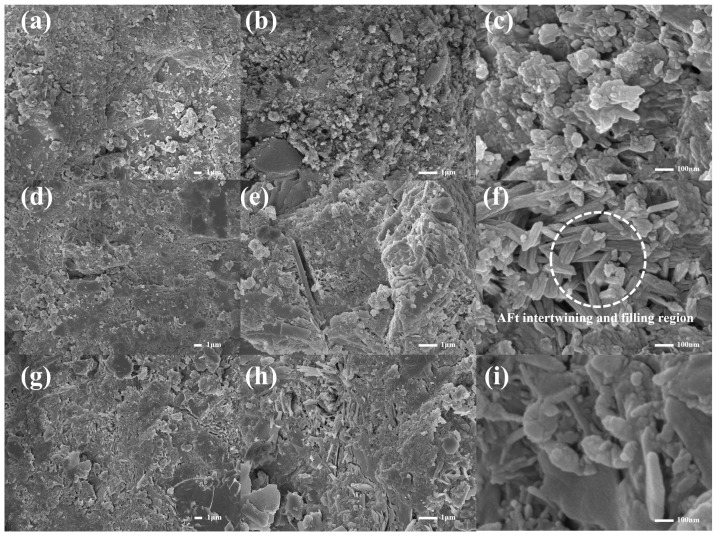
Microstructural morphology of cement paste at 28 days for different PMSO2 content: (**a**–**c**) PMSO2 content 0%; (**d**–**f**) PMSO2 content 0.1 wt%; and (**g**–**i**) PMSO2 content 0.2 wt%.

**Figure 19:**
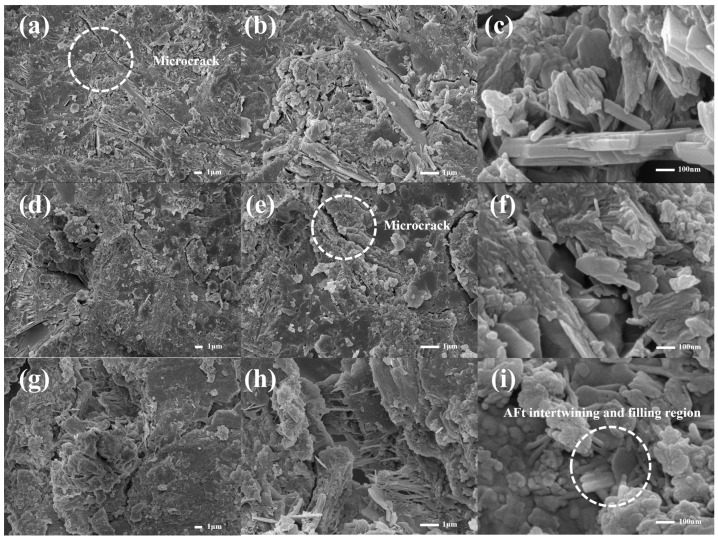
Microstructural morphology of cement paste at 28 days for different PMSO2 content: (**a**–**c**) PMSO2 content 0.3%; (**d**–**f**) PMSO2 content 0.4 wt%; and (**g**–**i**) PMSO2 content 0.5 wt%.

The microstructure and micro-morphology of cement paste at 28 days with PMSO2 micelle content of 0%, 0.1 wt%, 0.2 wt%, 0.3 wt%, 0.4 wt%, and 0.5 wt% are shown in Figure 18 and Figure 19. The primary products of cement hydration are Ca(OH)_2_, C–S–H, and AFt. Calcium hydroxide forms hexagonal crystals, calcium silicate hydrate exhibits elongated needle-like structures, while calcium silicate hydrate takes on fibrous forms [32]. As shown in Figure 18a–c, the cement paste structure in the control group appears distinctly loose, with numerous visible irregularly aggregated particles likely calcium hydroxide crystals and abundant pores, indicating insufficient overall density. Figure 18d–i reveal that at low PMSO2 micelle content, calcium hydroxide content slightly decreases while needle-like and fibrous materials significantly increase. These materials intertwine to fill pores, forming a denser structure. As shown in Figure 19a–i, higher PMSO2 micelle content increases the number of needle-like structures. The interlaced needle-like AFt hinders water exchange within the system [35]. Furthermore, with increasing PMSO2 content, noticeable cracks become visible, which also contributes to reduced strength. Combined with XRD data, the incorporation of PMSO2 influences the crystalline structure of cement hydration products. It provides time for the ordered growth of C–S–H gel and reduces the oriented crystallization of CH, thereby optimizing the pore structure and enhancing the system’s compactness [36].

#### 3.5.4. Contact Angle Analysis

**Figure 20:**
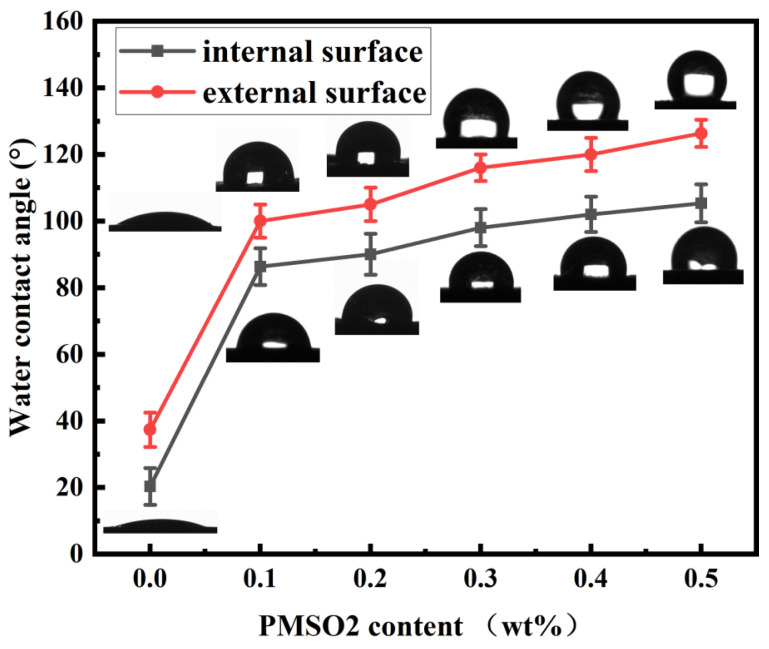
Effect of different PMSO2 content on the water contact angle of the inner and outer surfaces of PMSO2-modified cement mortar.

Figure 20 shows the effect of different PMSO2 content on the water contact angles of the inner and outer surfaces of PMSO2-modified cement mortar. The unmodified cement mortar exhibits hydrophilicity on both surfaces, with contact angles of 20° and 27° for the inner and outer surfaces, respectively. It can be observed that as the PMSO2 content increases, the contact angles of both the inner and outer surfaces of the modified cement mortar significantly increase. At a PMSO2 content of 0.1 wt%, the contact angles are 86° and 100°, respectively, indicating that the outer surface has achieved a hydrophobic state, while the inner contact angle also shows a marked increase compared to the control group. At a PMSO2 content of 0.5 wt%, the contact angles were 105° and 126°, respectively. The contact angles of diiodomethane and water were measured for the control mortar and the mortar modified with 0.5 wt% PMSO2. For the control mortar, the diiodomethane contact angle was 36° and the water contact angle was 27°, yielding a surface free energy of 71 mJ/m^2^. For the PMSO2-modified mortar (0.5 wt%), the diiodomethane contact angle was 89° and the water contact angle was 126°, giving a surface free energy of 13.1 mJ/m^2^. The control mortar exhibited high surface free energy, indicating hydrophilicity, whereas the PMSO2-modified mortar showed a significantly reduced surface free energy, demonstrating hydrophobicity. After cement hydration concludes and water is lost, micelles unfold, exposing their hydrophobic silicone chains externally. This forms a hydrophobic structure within the cement matrix. Overall, incorporating PMSO2 as a modifier into cement mortar enhances its hydrophobicity and achieves uniform dispersion throughout the mortar matrix.

#### 3.5.5. Porosity Analysis

**Figure 21:**
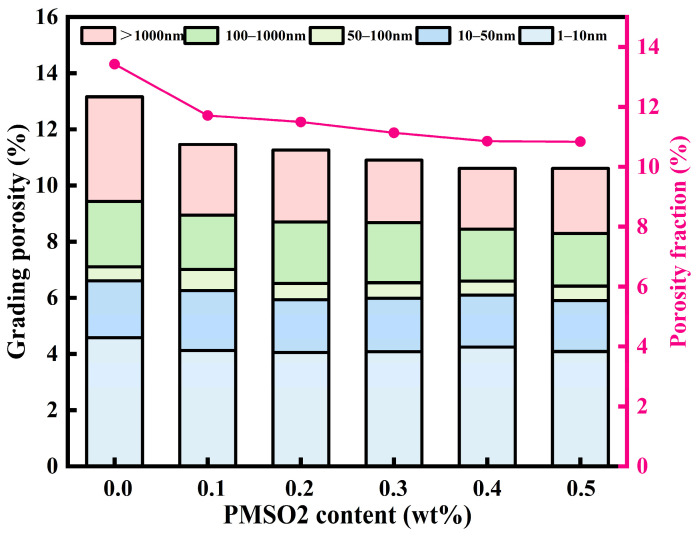
Pore volume and pore size distribution of mortar at different PMSO2 content.

The effect of PMSO2 micelles on the pore structure of cement mortar can be determined through low-field NMR testing. Based on the principle of classifying pores as harmless or harmful, the pores within concrete are categorized as follows: harmless pores (pore size < 20 nm), relatively harmless pores (pore size range 20–50 nm), harmful pores (pore size range 50–200 nm), and pores with a high concentration of harmful pores (pore size > 200 nm) [37]. As shown in Figure 21, the porosity of the control group was 13.43%. The porosities for PMSO2 content of 0.1–0.5 wt% were 11.71%, 11.49%, 11.13%, 10.85%, and 10.83%, respectively. The porosity initially decreased and then increased with increasing PMSO2 content. However, the overall porosity remained lower than the control value, indicating that adding PMSO2 micelles to cement reduces the porosity of cement mortar, resulting in a denser structure. This also confirms that incorporating PMSO2 micelles into cement reduces the water absorption rate of cement mortar. As shown in Figure 21, the distribution of harmful pore sizes exceeding 50 nm at PMSO2 content levels of 0.1–0.5 wt% was 6.55%, 5.20%, 5.33%, 4.92%, 4.51%, and 4.71%, respectively. At low PMSO2 content, the number of harmful pores significantly decreases, indicating that PMSO2 micelles at low concentrations reduce the number of large pores, resulting in a denser structure. This directly correlates with the strength increase observed at low PMSO2 content. However, as PMSO2 content increases, the number of large pores also rises. The changes in pore size distribution confirm the previously mentioned decrease in compressive strength at high PMSO2 content.

#### 3.5.6. Mechanism of PMSO Micelle Action

**Figure 22:**
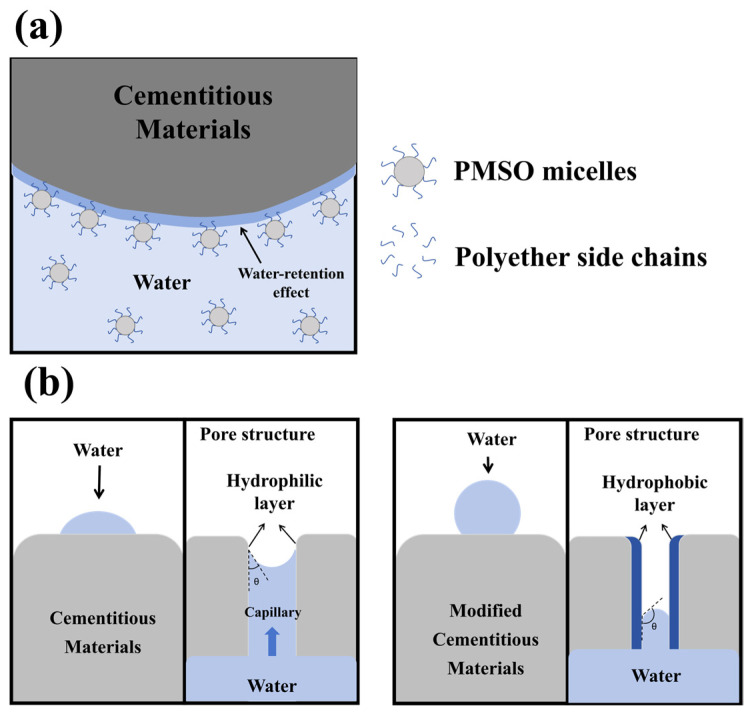
(**a**) Mechanism of action of amphiphilic polyether-modified silicone oil micelles in cementitious materials; (**b**) schematic illustration of the pore structure effect.

The essence of micelle formation is the self-assembly of amphiphilic PMSO molecules in water: the hydrophobic polysiloxane backbone is encapsulated inside, while the hydrophilic polyether segments form aggregates in water. The micelle size is related to the HLB value; the higher the HLB value, the more hydrophilic segments there are, the better the dispersion in water, and the smaller the micelle size [38,39]. The addition of polyether-modified silicone oil micelles facilitates cement hydration at the middle and later stages. A proposed mechanism for this phenomenon is illustrated in Figure 22a. Amphiphilic polymers possess surface activity and can reduce surface and interfacial tension. The hydrophilic polyether segments on the surface of the micelles dispersed in water adsorb onto cement particles via hydrogen bonding and van der Waals forces. These hydrophilic chains impose a low barrier to water transport, thus having little impact on early-stage hydration. Furthermore, the hydrophilic polyether segments adsorbed on cement particles exhibit water-retention properties, which benefit hydration at the middle and later stages. In addition, the adsorption of micelles onto cement particles enhances their stability, leading to smaller hydration products and a denser microstructure. However, at a high dosage of polyether-modified silicone oil micelles, excessive adsorption on the cement surface may adversely affect cement hydration. Figure 22b is a schematic illustration of the pore structure effect. Since the polymer we prepared is in a liquid state, the polyether segments of the polymer adsorb onto the pore surfaces, rendering the pores hydrophobic and reducing the capillary flow effect. The adsorbed layer on the capillary pore walls cannot provide sufficient capillary rise force for liquid water, thereby significantly reducing water absorption and retention [40]. The wettability of the pores is fundamentally altered, thereby suppressing water and ion transport.

### 3.6. Effect of Different PMSO2 Content on Concrete

#### 3.6.1. Concrete Water Absorption Rate

**Figure 23:**
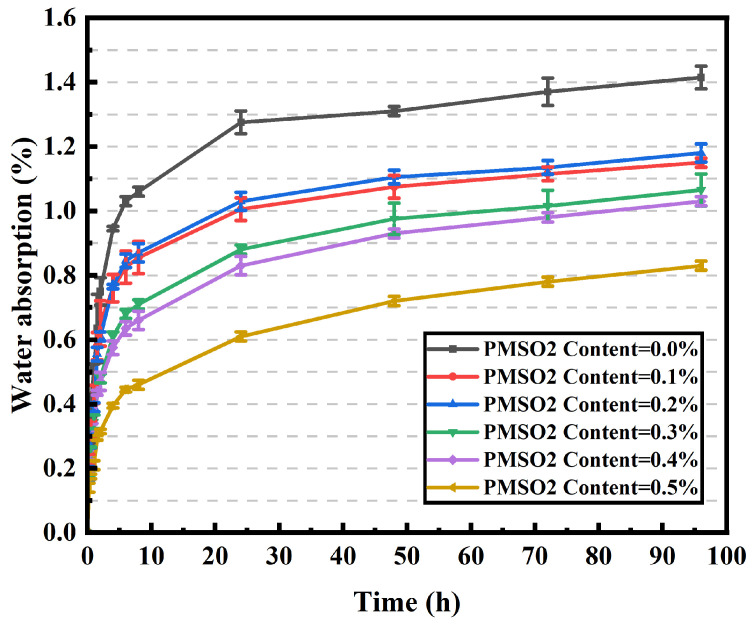
Water absorption of concrete specimens with different PMSO2 content.

The water absorption rates of concrete with different PMSO2 content are shown in Figure 23. As the PMSO2 content increases, the water absorption rate of concrete specimens gradually decreases relative to the control group, and the rate of decrease slows over time. The water absorption rates at 48 h for the control group and the 0.5 wt% PMSO2 group were 1.31% and 0.72%, respectively, representing a 45.04% reduction. This demonstrates that the addition of PMSO2 significantly reduces the water absorption rate of concrete. The water absorption reduction rate of concrete is 45.04%, lower than that of mortar at 50.27%, which is attributed to the complex mixture composition of concrete. Concrete contains mineral powders, fly ash, and aggregates (sand/stone), which adsorb portions of PMSO2 aggregates and reduce their effective concentration within the cement matrix. Furthermore, the formation of additional capillaries at the aggregate–cement interface weakens the hydrophobic barrier effect, meaning aggregate addition diminishes hydrophobic modification efficiency [19].

#### 3.6.2. Resistance to Chloride Ion Permeation

**Figure 24:**
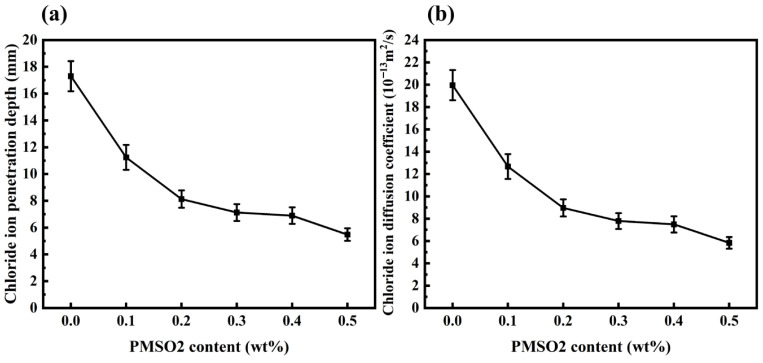
(**a**) Chloride ion penetration depth of concrete specimens with different PMSO2 content; (**b**) chloride ion diffusion coefficient of concrete specimens with different PMSO2 content.

The durability of concrete is closely related to its permeability to water and other harmful substances. The permeability of chloride ions in concrete reflects both the concrete’s resistance to permeation and its ability to resist reinforcement corrosion [41]. Concrete with higher permeability allows faster penetration of gases, liquids and other aggressive substances such as chloride, resulting in rapid corrosion of the reinforcing steel [42]. The effects of different PMSO2 content on the chloride ion penetration depth and diffusion coefficient in concrete are shown in Figure 24. The control group exhibited a chloride ion penetration depth of 17.3 mm and a diffusion coefficient of 19.96 × 10^−13^ m^2^/s. At a PMSO2 content of 0.5 wt%, the chloride penetration depth decreased to 5.5 mm, with a diffusion coefficient of 5.83 × 10^−13^ m^2^/s and 70.8% reduction. These results demonstrate that PMSO2 addition significantly enhances concrete’s resistance to chloride ion penetration. Higher PMSO2 content results in reduced chloride penetration depth and lower chloride diffusion coefficients in concrete. The enhanced resistance to chloride ion penetration is primarily attributed to the hydrophobic modification effect of PMSO2 within the concrete matrix. When PMSO2 is incorporated into concrete, its polyether segments adsorb onto pore surfaces, imparting hydrophobicity to the pores. This slows capillary flow effects, enhances the overall hydrophobicity of the concrete matrix, and retards the migration of polar substances such as water and chloride ions, thereby reducing water and chloride ion permeation. The denser the pore structure of cement-based materials and the smaller the proportion of connected pores, the lower the chloride diffusion coefficient [43,44]. Consequently, incorporating PMSO2 into concrete improves its permeability resistance and corrosion protection for reinforcing steel.

#### 3.6.3. Corrosion Resistance

**Figure 25:**
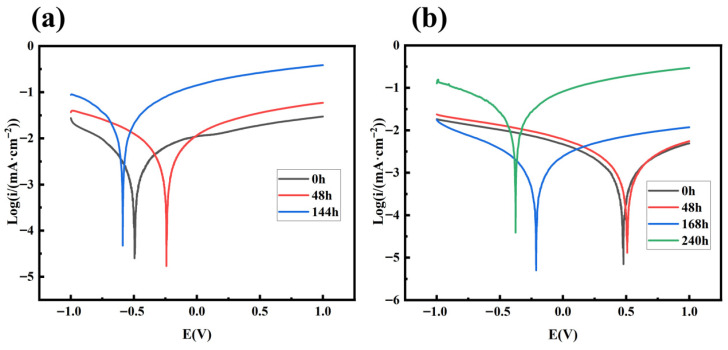
Tafel polarization curves of embedded steel bars in different specimens: (**a**) control specimen; (**b**) specimen with 0.5 wt% PMSO2 content.

**Figure 26:**
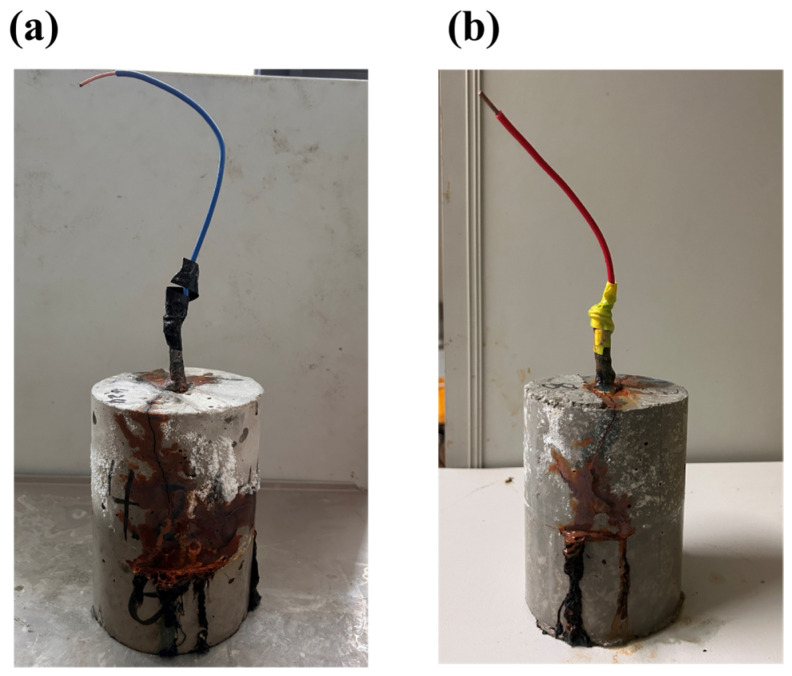
Photographs of cracked embedded steel bars in different specimens: (**a**) control specimen; (**b**) specimen with 0.5 wt% PMSO2 admixture.

The Tafel polarization curves of PMSO2 concrete specimens at different time points are shown in Figure 25. The dispersion observed in these curve results is primarily attributed to the complex electrochemical system formed during the corrosion process. When calculating the Tafel slope constant, particular attention should be paid to the slope within the linear region of the curve [45]. The corrosion current density (i_corr_) was obtained by determining the intersection point of the Tafel curve slope, with specific data presented in Table 10. The data reveal that the corrosion potential measured in the control group at the initial state is negative, indicating that the passivation film on the steel bars immersed in 5% NaCl solution had already suffered localized damage due to chloride ion intrusion prior to accelerated corrosion. During the initial corrosion phase, the polarization curves of the experimental group exhibited similarity, with corrosion potentials stabilizing near 0.5 V. The elevated corrosion potential indicates a dense and intact passivation film on the steel bar surface, demonstrating excellent corrosion resistance. As accelerated corrosion time increased, the polarization curves shifted negatively, and the corrosion current density rose [46]. The control group experienced a sharp increase in corrosion current density at 48 h, indicating severe corrosion of the steel bars. Both the corrosion potential and corrosion current density of the experimental group exceeded those of the control group. Generally, a lower i_corr_ value indicates a slower corrosion rate and stronger corrosion resistance [47]. Figure 26 shows actual photographs of cracked embedded steel bars in different specimens. The concrete incorporating PMSO2 exhibited corrosion cracking at 240 h, significantly later than the 144 h cracking time observed in the control group. This demonstrates that polyether-modified silicone oil micelles form a barrier by adsorbing onto concrete capillary pore walls via their hydrophobic components and refining the pore structure. This barrier significantly impedes the transport of chloride ions and oxygen to the reinforcement surface, thereby greatly enhancing the corrosion resistance of reinforced concrete.

## 4. Conclusions

In this study, a series of amphiphilic polyether-modified silicone oils (PMSOs) with different HLB values were synthesized via a silane-hydrogenation reaction. Their self-emulsified micelles were used as hydrophobic polymers for internal admixture in cement-based materials. This study investigated the effects of amphiphilic polymers on the hydrophobicity, water resistance, mechanical properties, microstructure, and corrosion resistance of cement-based materials. The main conclusions are as follows:

(1) Amphiphilic polyether-modified silicone oil (PMSO) polymers with different HLB values were synthesized via a silyl-hydrogen addition reaction. Infrared spectroscopy and nuclear magnetic resonance (NMR) hydrogen spectroscopy confirmed that the polyether segments were grafted onto the siloxane backbone via Si-C bonds. Amphiphilic polyether-modified silicone oil polymers were capable of self-emulsification to form stable micelles. The micelle diameter decreased from 161.4 nm to 79.9 nm as the HLB value increased, and no phase separation or precipitation occurred during three months of storage at room temperature.
(2) PMSO micelles with different HLB values all reduced the water absorption rate of cement mortar, with PMSO2 (HLB = 4) showing the best performance: at a dosage of 0.5 wt%, the water absorption rate after 48 h decreased by 50.27% compared to the control group. In terms of mechanical properties, at a low dosage of 0.1 wt%, both PMSO1 and PMSO2 improved flexural and compressive strengths. At 28 days, when PMSO2 was dosed at 0.1 wt%, the flexural and compressive strengths increased by 12.50% and 14.19%, respectively, compared to the control group. The system with the best overall performance was PMSO2.
(3) The addition of PMSO2 reduced the standard consistency water requirement of the cement paste, prolonged the initial and final setting times, and improved the workability of the mortar. XRD and SEM analyses indicated that PMSO2 did not alter the types of hydration products; however, at low dosages, it promoted the formation of acicular AFt and fibrous C–S–H, which filled pores and enhanced density; at high dosages, microcracks appeared. Low-field NMR results show that PMSO2 reduced the total porosity of the mortar from 13.43% to 10.83%, and the number of harmful pores larger than 50 nm decreased significantly.
(4) After modification with PMSO2, the water contact angles on the inner and outer surfaces of the mortar reached 105° and 126°, respectively, demonstrating hydrophobicity. Forty-eight hours after modification, the water absorption rate of the concrete decreased by 45.04%, and the chloride ion diffusion coefficient decreased by 70.8%. Electrochemical accelerated-corrosion tests showed that the time to cracking of steel bars in PMSO2-modified concrete was extended from 144 h in the control group to 240 h, significantly improving the corrosion resistance of the reinforced concrete.
(5) The limitations of this study include: the corrosion tests were based solely on accelerated electrochemical methods, without long-term natural exposure experiments; and the micelle characterization was carried out in deionized water, without verification in real cement pore solution. Future work will include long-term field exposure tests and optimization studies of micelle characterization in cement pore solution.

## Figures and Tables

**Table 1:** Chemical composition of reference cement, mineral powder, and fly ash.

Chemical Composition (%)	SiO_2_	Al_2_O_3_	Fe_2_O_3_	CaO	MgO	SO_3_	Na_2_O
Reference cement	20.16	4.68	3.51	62.49	4.04	2.30	0.71
Mineral powder	30.85	15.14	0.37	40.94	7.39	2.54	0.42
Fly ash	46.04	31.1	6.16	6.65	0.95	1.36	1.27

**Table 2:** Cement net slurry formulation.

Samples	Cement (g)	Water (g)	PMSO Micelles (g)
Control	450	145	0
1P	450	143	2.5
2P	450	141	5
3P	450	139	7.5
4P	450	137	10
5P	450	135	12.5

**Table 3:** Cement mortar formulation.

Samples	Sand (g)	Cement (g)	Water (g)	PMSO Micelles (g)
Control	1350	450	198	0
1P	1350	450	196.2	2.25
2P	1350	450	194.4	4.5
3P	1350	450	192.6	6.75
4P	1350	450	190.8	9
5P	1350	450	189	11.25

**Table 4:** Concrete mix design.

Materials (Kg/m^3^)	Water	Cement	Mineral Powder	Fly Ash	Sand	Stone	P900-10%	Additives	PMSO Micelles
Control	131.36	147	191	128	785	1060	8.5	8.5	0
1CP	129.66	147	191	128	785	1060	8.5	8.5	2.125
2CP	127.96	147	191	128	785	1060	8.5	8.5	4.25
3CP	126.26	147	191	128	785	1060	8.5	8.5	6.375
4CP	124.56	147	191	128	785	1060	8.5	8.5	8.5
5CP	122.86	147	191	128	785	1060	8.5	8.5	10.625

**Table 5:** Synthesis and molecular weight measurement of amphiphilic polyether-modified silicone oil polymers.

Samples	HLB_th_	Molar Ratio of Polyether to Hydrogen-Containing Silicone Oil	State of the Compound	Mn ^a^ (g/mol)	Mw ^b^ (g/mol)	PDI
PMSO0	3	1/6.3	Turbid state	/	/	/
PMSO1	3.5	1/5.0	Transparent and uniform	2378	11,200	4.71
PMSO2	4	1/4.0	Transparent and uniform	2435	11,116	4.56
PMSO3	4.5	1/3.4	Transparent and uniform	3546	11,134	3.14
PMSO4	5	1/2.7	Transparent and uniform	4438	10,117	2.28

^a^ Number average molecular weight; ^b^ weight average molecular weight.

**Table 6:** Comparison of design values and measured values for polyether-modified silicone oils with different HLB values.

Samples	HLB_th_	Volume of Turbid Water (mL)	HLB_exp_
PMSO1	3.5	3.9	3.81
PMSO2	4	4.1	4.32
PMSO3	4.5	4.3	4.82
PMSO4	5	4.6	5.51

**Table 7:** Silane-hydrogen bond conversion efficiency of polyether-modified silicone oils with different HLB values.

HLB_th_	Theoretical Conversion Rate (%)	Actual Conversion Rate (%)
3.5	19.94	22.26
4	24.70	26.29
4.5	30.30	35.19
5	37.04	45.86

**Table 8:** Appearance and particle size of micelles in polyether-modified silicone oil with different HLB values.

HLB_th_	Micelle Morphology	Micelle Size (nm)	PDI
3.5	white liquid	161.4	0.186
4	light white liquid	150.9	0.129
4.5	light white liquid	121.9	0.095
5	light blue liquid	79.9	0.063

**Table 9:** Particle size of different PMSO micelles in NaCl solutions with increasing concentration.

Samples	NaCl Concentration (mol/L)	Micelle Size (nm)	PDI
PMSO1	0.1	163.8	0.213
	0.2	160.4	0.182
	0.5	162.1	0.197
	1	164.3	0.201
PMSO2	0.1	148.5	0.213
	0.2	150.4	0.156
	0.5	153.6	0.113
	1	151.7	0.148
PMSO3	0.1	122.2	0.162
	0.2	123.7	0.139
	0.5	126.1	0.146
	1	126.6	0.122
PMSO4	0.1	81.6	0.114
	0.2	78.1	0.103
	0.5	79.5	0.098
	1	83.7	0.074

**Table 10:** Corrosion current density and corrosion voltage of embedded reinforcing bars in different specimens.

Samples	Time (h)	Corrosion Potential (V)	Corrosion Current Density (i_corr_ (mA·cm^−2^))
Control	0	−0.495	8.28 × 10^−5^
	48	−0.394	1.25 × 10^−3^
	144	−0.588	5.13 × 10^−3^
Experimental Group	0	0.478	2.36 × 10^−5^
	48	0.508	2.98 × 10^−5^
	168	−0.214	3.06 × 10^−5^
	240	−0.376	5.69 × 10^−3^

## Data Availability

Data are contained within the article.

## Supplementary Materials

The following are available online at https://www.mdpi.com/article/10.3390/polym18101153/s1, Table S1: A summary of studies on integral hydrophobic modified cementitious materials.
