# Supplementary material for: Synthesis of Amphiphilic Polyether-Modified Silicone Oil Polymers and the Application of Their Micelles in Enhancing the Overall Waterproofing and Corrosion Resistance of Cement-Based Concrete Materials
Source: Polymers (Basel). 2026 May 8;18(10):1153. doi: 10.3390/polym18101153 (PMC13210907; doi:10.3390/polym18101153)
Supplement: Supplementary file 1 [file polymers-18-01153-s001.zip › polymers-4280690-supplementary.pdf]

Supporting Information

Synthesis of amphiphilic polyether-modified silicone oil polymers and the application of their micelles in enhancing the overall waterproofing and corrosion resistance of cement-based concrete materials

Yujie Luo [1], Fen Zhou [1], Shuangping Ma [1], Depeng Gong [1], Zhanbo Wang [1], Xi Li[2] and Chaocan Zhang [1,*]

Table S1. A summary of studies on integral hydrophobic modified cementitious materials.

Hydrophobic system	Emulsifier required	Effect on hydration	Effect on mechanical strength	Effect on water absorption
Silane emulsion [1]	Yes	Slightly inhibited	Slightly decreased (compressive strength reduced)	Water absorption significantly reduced
Calcium stearate emulsion [2]	Yes	Inhibited	At 1.2 wt% dosage, compressive strength decreased by 14.96%, flexural strength decreased by 13.95%	At 1.2 wt% dosage, water absorption at 48 h reduced by 51.22%
Paraffin emulsion [3]	Yes	Inhibited	At 2% dosage, compressive strength decreased by 10.86%	Water absorption reduced by 58.3%
Silane emulsion + silica fume [4]	Yes	Inhibited	Significantly decreased (compressive strength reduced by 36.15%)	Water absorption coefficient reduced by 59.22%
Polymer latex (SBR) [5]	Yes	May delay	Increases at low dosage, decreases at high dosage	Water absorption reduced
PMSO micelles (this work)	No	Not inhibited (promotes later-age hydration)	At 0.1 wt% dosage, flexural strength increased by 12.5%, compressive strength increased by 14.19%	At 0.5 wt% dosage, water absorption of mortar reduced by 50.27%

[1] Zhang, C.; Zhang, S.; Yu, J.; Kong, X. Water absorption behavior of hydrophobized concrete using silane emulsion as admixture. *Cement and Concrete Research* **2022**, *154*, 106738, doi:10.1016/j.cemconres.2022.106738.
[2] Wu, S.; Zhang, C.; Zhou, F.; Ma, S.; Zheng, H. The effect of nano-scale calcium stearate emulsion on the integral waterproof performance and chloride resistance of cement mortar. *Construction and Building Materials* **2022**, *317*, 125903, doi:10.1016/j.conbuildmat.2021.125903.

[3] Zhou, Y.; Liao, X.; Li, L.; Guo, M.; Hu, B. Using nonionic paraffin emulsion to make waterproof engineered cementitious composites: mechanical properties and hydrophobic performance. *Construction and Building Materials* **2024**, *428*, 136222, doi:10.1016/j.conbuildmat.2024.136222.
[4] Song, P.; Qiu, H.; Wang, X.; Wang, L.; Liu, R.; Tang, K.; Zhou, J. Hydrophobic anti-icing cement mortar: Synergistic effects of silane emulsion and ammonium carboxylate solution with silica fume. *Construction and Building Materials* **2025**, *498*, 144026, doi:10.1016/j.conbuildmat.2025.144026.
[5] Borhan, T.M.; Al Karawi, R.J. Experimental investigations on polymer modified pervious concrete. *Case Studies in Construction Materials* **2020**, *12*, e00335, doi:10.1016/j.cscm.2020.e00335.
